# Fads and Fallacies in Psychiatry

**DOI:** 10.1192/pb.bp.114.047522

**Published:** 2014-12

**Authors:** Philip Timms

**Figure:**
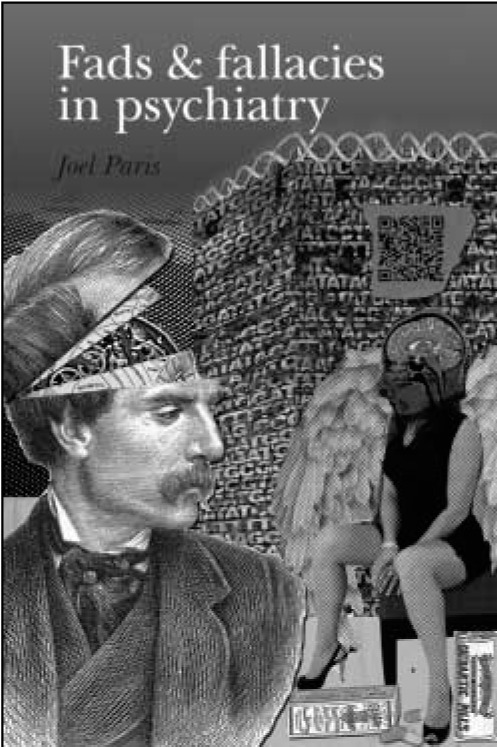


Joel Paris is a veteran professor of psychiatry in Canada. He examines our periodic enthusiasms for unfounded ideas and the ‘cognitive errors of wishful thinking’ that underpin them. Perhaps to reassure us that psychiatry is not uniquely gullible, he starts with a short chapter on fads in general medicine. They can be seen to be driven by lack of evidence, therapeutic optimism and the power of the charismatic teacher. He then looks at how these have influenced psychiatry. Although, to my mind, he is too dismissive of sociology, he is generally an even-handed critic. He scrutinises the foundations of aetiology, genetics, epidemiology and diagnosis, then the areas of intervention, psychopharmacology, psychotherapy, and prevention.

Some of the offences he describes are historical, such as the uncritical adoption of electroconvulsive therapy (ECT) and psychosurgery. Topical issues include the outside influence of drug companies and their wilful concealment of important negative findings. Inside psychiatry, academic psychiatrists are taken to task. The understandable desire for precision has led to diagnostic inflation into ever more categories (thank you, DSM). Low thresholds for disease intensity threaten to define millions more as ‘cases’.

Although he addresses psychiatry as a whole, his outlook is inevitably framed by current issues in North American psychiatry. Many of us in the UK National Health Service seldom see anyone without a psychosis, so diagnostic inflation may seem somewhat academic. Some North American notions such as ‘hospital privileges’ need explanation. And, in the UK at least, quetiapine is not, as yet, ‘the new Valium’. I blame the editors.

Who is this book for? It is too sober and scholarly for a general readership, which will expect tales of fiendish experiments and outlandish treatments. The voice is often that of a valedictory address, which may be too personal for the more academic. This book could most benefit the new entrant to psychiatry, pulled this way and that by fashion, optimism and authority. It could help to balance their necessary (and healthy) therapeutic optimism with a corrective evidence-based scepticism. And embolden the tyro to challenge the teacher, however authoritative, charming or charismatic that teacher might be.

